# Cellular senescence in skeletal disease: mechanisms and treatment

**DOI:** 10.1186/s11658-023-00501-5

**Published:** 2023-10-27

**Authors:** Xu He, Wei Hu, Yuanshu Zhang, Mimi Chen, Yicheng Ding, Huilin Yang, Fan He, Qiaoli Gu, Qin Shi

**Affiliations:** 1 Department of Orthopedics, The First Affiliated Hospital of Soochow University, Orthopedic Institute of Soochow University, Medical College of Soochow University, 899 Pinghai Road, Suzhou, Jiangsu 215031 People’s Republic of China; 2 Department of Orthopedics, Wuxi Ninth People’s Hospital Affiliated to Soochow University, Wuxi, Jiangsu 214026 People’s Republic of China; 3 Department of Orthopedics, Children Hospital of Soochow University, No. 92 Zhongnan Street, Suzhou, Jiangsu 215000 People’s Republic of China; 4 Xuzhou Medical University, 209 Copper Mountain Road, Xuzhou, 221004 People’s Republic of China

**Keywords:** Cellular senescence, Chronic inflammation, Age-related orthopaedic diseases, Signalling pathways, Bone marrow

## Abstract

The musculoskeletal system supports the movement of the entire body and provides blood production while acting as an endocrine organ. With aging, the balance of bone homeostasis is disrupted, leading to bone loss and degenerative diseases, such as osteoporosis, osteoarthritis, and intervertebral disc degeneration. Skeletal diseases have a profound impact on the motor and cognitive abilities of the elderly, thus creating a major challenge for both global health and the economy. Cellular senescence is caused by various genotoxic stressors and results in permanent cell cycle arrest, which is considered to be the underlying mechanism of aging. During aging, senescent cells (SnCs) tend to aggregate in the bone and trigger chronic inflammation by releasing senescence-associated secretory phenotypic factors. Multiple signalling pathways are involved in regulating cellular senescence in bone and bone marrow microenvironments. Targeted SnCs alleviate age-related degenerative diseases. However, the association between senescence and age-related diseases remains unclear. This review summarises the fundamental role of senescence in age-related skeletal diseases, highlights the signalling pathways that mediate senescence, and discusses potential therapeutic strategies for targeting SnCs.

**Graphical Abstract:**

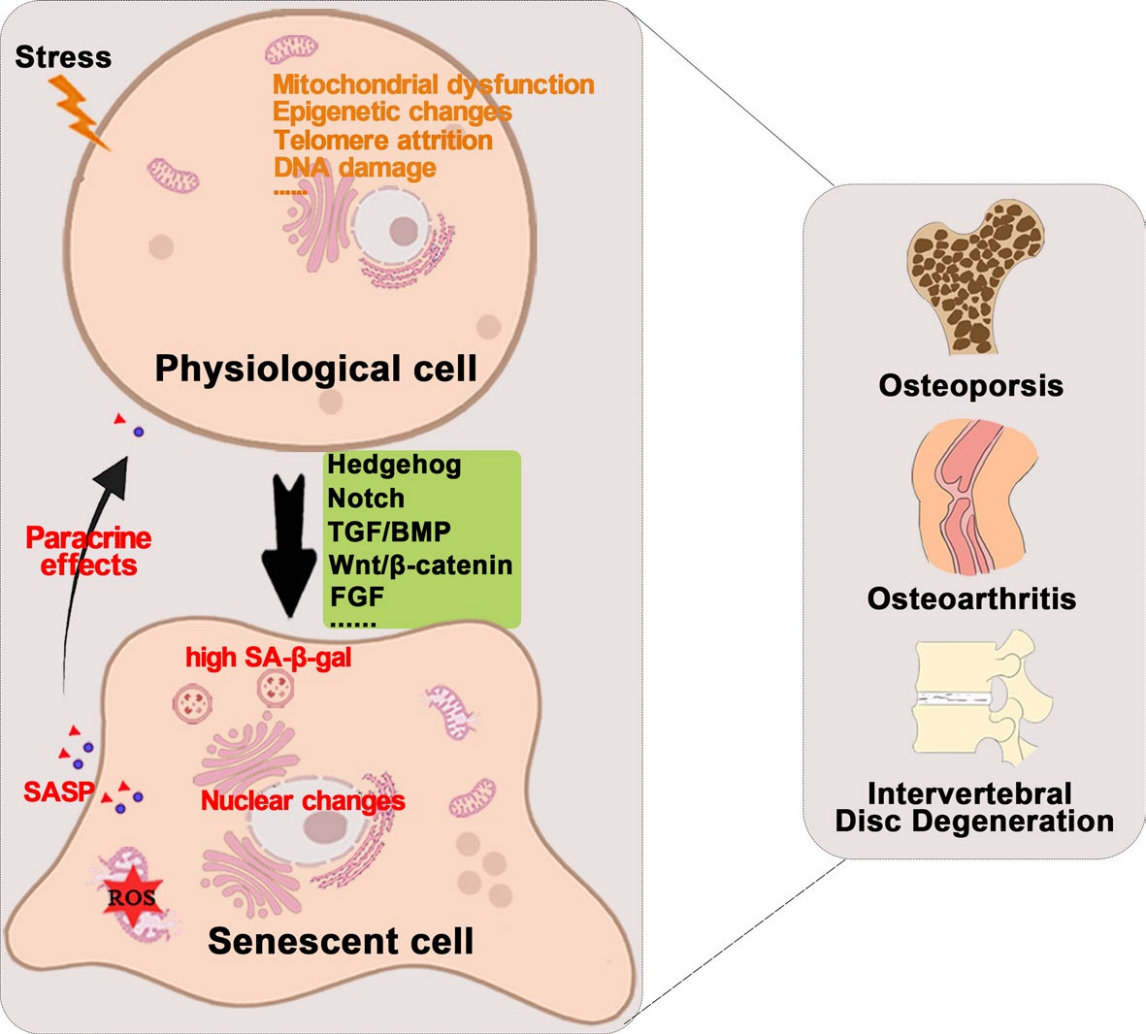

## Introduction

Age-related musculoskeletal diseases such as osteoporosis (OP), osteoarthritis (OA), and intervertebral disc degeneration (IDD) critically affect the motor functions and quality of life of elderly individuals (1). Unfortunately, although various drugs, (such as bisphosphonates, recombinant human parathyroid hormone, denosumab for OP, and paracetamol for OA), have been licenced, their benefits are limited due to side effects or the poor overall health of elderly individuals (2, 3). Aging involves complex mechanisms, including genetic mutations, telomere shortening, epigenetic alterations, protein deformation, mitochondrial damage, and cellular senescence, which are responsible for the onset of age-related diseases (4). Thus, investigating and manipulating the mechanisms underlying aging are important future research goals. Among the fundamental mechanisms mentioned above, cellular senescence has received considerable attention in recent years.

Cellular senescence refers to the stable condition of cell cycle arrest, first described by Hayflick and Moorhead in the early 1960s. Senescent cells (SnCs) are produced in the early stages of embryonic development and accumulate with age (5). However, SnCs exert deleterious effects on tissues by secreting a plethora of inflammatory cytokines, chemokines, oxidative stress-related proteins, growth factors, and proteases, which is termed the senescence-associated secretory phenotype (SASP). Accumulated SnCs are a hallmark of aging and contribute to age-related diseases, including OP, diabetes, and Alzheimer's disease (6). Interestingly, SnCs have dual effects on tumour development, which may depend on the immune microenvironment or cell cycle stage, as cell cycle arrest is helpful for tumour suppression (7).

The skeletal system consists of bones, joints, cartilage tissues, and ligaments that work together to maintain homeostasis of the motor system and the internal environment (8). Bone remodeling occurs throughout life. Bone tissue comprises four types of cells: osteoblasts, osteoclasts, osteocytes, and osteoprogenitor (9). They undergo fundamental changes during the aging process (10). Senescent mesenchymal stem cells (MSCs) exhibit decreased osteogenesis and increased adipogenesis, moreover, senescent osteocytes or osteoclasts produce SASP (10). However, the underlying mechanisms by which SnCs and SASP regulate bone remodelling and induce disease are still under investigation.

In this review, we summarise the role of senescence in the skeletal system, discuss its underlying mechanisms, and propose new strategies for treating age-related diseases by targeting senescence.

## Characteristics of cellular senescence

Senescence was originally described as a consequence of telomeric dysfunction. Physiologically, telomeres shorten during cell division. After approximately 50–70 divisions, telomeres lose their protective structure, consequently activating tumour suppressor genes and cell cycle inhibitors, thus leading to cell growth inhibition. This phenomenon is termed replicative senescence or Hayflick limit (11). Senescence is an innate response triggered by various stimuli such as epigenetic changes, DNA damage, telomere attrition, and mitochondrial malfunction (12).

*Epigenetic changes:* Epigenetics mainly involve DNA methylation, post-translational modification of histones, and chromatin remodeling. DNA methylation patterns are primarily established and maintained by three DNA methyltransferases that ensure high fidelity of the DNA methylation patterns inherited by mammals after each cell division (13). Histone modifications are covalent post-translational modifications, including acetylation, methylation, phosphorylation, and ubiquitination. DNA methyltransferases, histone methylases, demethylases, acetylases, and deacetylase-related protein complex enzyme systems work together to maintain epigenetic patterns (14). Substantial evidence has linked epigenetic changes to aging. Histone demethylases regulate the lifespan of caenorhabditis elegans by targeting the insulin/IGF-1 signalling pathway (15). Downregulation of histone deacetylases 2 and 7 in a replicative aging model cause senescence of dermal fibroblasts (16). Additionally, inhibition of E1A binding protein expression during aging promotes cellular senescence in human umbilical cord-derived MSCs via the p53/p21 pathway (17).

*DNA damage:* Senescence and accumulation of SnCs are associated with the cellular DNA damage response (DDR). Prolonged exposure to single-stranded DNA and generation of DNA double-strand breaks can develop DDR. The emergence of the DDR activates the p53/p21 and p16 axes, thereby triggering cell cycle arrest and leading to cellular senescence (12). Werner syndrome (WS) and Hutchinson-Gilford progeria syndrome (HGPS), the two most characteristic forms of human progeria, are closely associated with an altered DDR response (18). After induction by DNA double-strand breaks, mouse hepatocytes exhibited distinct senescence phenotypes including senescence pathology, senescence markers, fused mitochondria, and altered gene expression profiles (19).

*Telomere attrition:* Telomeres found at the ends of chromosomes are highly conserved tandem TTAGGG nucleotide repeats. These sequences form a "cap structure" that maintains the integrity of the chromosome. Telomerase, a reverse transcriptase, plays a crucial role in telomere maintenance by counteracting telomere shortening (20). Thus, telomere depletion is a plausible explanation for replicative senescence (11, 21). Importantly, telomere shortening has been observed in normally aging humans and mice (22). Telomere dysfunction is closely associated with aging and age-related diseases, including aplastic anaemia, idiopathic pulmonary fibrosis, chronic obstructive pulmonary disease, nonalcoholic fatty liver disease, type 2 diabetes, heart disease, atherosclerosis, OP, and OA (23).

*Mitochondrial dysfunction:* Mitochondria are essential organelles in the oxidative respiratory chain in vivo. Mitochondrial dysfunction drives cellular senescence in several ways. Mice with mutations in mitochondrial DNA develop multiple age-related phenotypes, including thymus involution, testicular atrophy associated with spermatogonia depletion, decreased bone mass, decreased intestinal crypts, and weight loss (24); Mitochondrial phosphatase 5 deficiency in the inner mitochondrial membrane promotes cellular senescence by activating the mammalian target of rapamycin (mTOR) signalling pathways (25). Studies have shown that Mitochondrial dysfunction induces a unique SASP-like senescence phenotype called mitochondrial dysfunction-associated senescence (MiDAS). Cells in an MiDAS environment have a lower NAD + /NADH ratio (26). Mitochondrial dysfunction also increases intracellular reactive oxygen species (ROS) levels, leading to aging (27, 28).

Morphologically, SnCs exhibit a broad, flattened shape and share similar biochemical characteristics such as G1 phase arrest in the cell cycle, elevated β-galactosidase activity, loss of laminin B1 (nuclear changes), and impaired mitochondria that generate excessive ROS (29). The enzymatic activity of senescence-associated β-galactosidase (SA-β-gal) is related to the increased content of lysosomes (29). SA-β-gal-targeted prodrugs selectively eliminate SnCs. Inactivation of p53 or p16^INK4a^ enables cells to escape growth arrest, leading to prolonged lifespan (30). Thus, the combination of SA-β-gal activity and p21, p53, p16, and γH2AX expression is a major index for SnCs identification.

## Senescence and aging

Aging is a multifaceted degenerative process that results in systemic alterations and has been implicated in the pathogenesis of numerous chronic diseases. Factors involved in aging include cellular senescence, genetic variability, telomere shortening, impaired stem cell function, and excessive ROS production (12). However, it remains unclear whether senescence is a consequence of aging. p16 is a senescence marker, and the number of p16^Ink4a^-expressing cells progressively increases with age. The removal of p16^Ink4a^-positive SnCs can delay or alleviate several age-related diseases. In contrast, the transplantation of SnCs into young animals causes age-related disorders (31). Elderly individuals develop a low-level chronic inflammation, referred to as immune aging, which is possibly induced by SnCs accumulation (32). Although the mechanisms contributing to SnCs accumulation remain unknown, available evidence suggests that SnCs accumulate in aged tissues and are critical triggers of degenerative diseases (33). P21, as a critical cell cycle regulatory molecule, is an essential mediator of p53-dependent cell cycle arrest and has been extensively studied in recent years (34). P21 is highly expressed in the visceral adipose tissue of mice with high-fat diet induced diabetes mellitus, and intermittent elimination of cells with high p21 expression in visceral adipose tissue ameliorates insulin resistance in obese mice (35). During aging, the expression of p21 and p16 increases in mouse muscle tissues, and high expression of p21 is predominantly found in muscle fibres (36). High p21 expression induces skeletal muscle senescence and SASP secretion in mice, which in turn leads to lower body weight and reduced skeletal muscle weight (37). Surprisingly, p16 and p21, two cellular markers of senescent cells, have distinct expression populations; for example, p16 is highly expressed in senescent pancreatic islet tissue, whereas p21 is highly expressed in adipose tissue (35, 38). The relative contribution of each pathway to senescence induction is unknown, and therefore, their role in cellular senescence requires further investigation.

MicroRNAs (miRNAs) are short non-coding RNAs consisting of approximately 20–25 nucleotides bound to the 3' untranslated region of messenger RNA that subsequently inhibit the transcription-translation of target genes (39, 40). In a mouse HGPS model, miR-29 upregulation occurred in a p53-dependent manner, and with normal aging in mice, miR-29 expression was upregulated (41). MiR-181a is one of the most abundant miRNAs in lymphocytes and is essential for T-cell senescence regulation (42). With age, reduced miR-181a levels impair T-cell functions (43, 44). MiR-181a-5p was downregulated in hippocampal neural stem cells from senescent mice and was involved in hippocampal-dependent learning and memory deficits associated with aging (45). Therefore, miRNAs mediate the onset of aging, and their roles and characteristics in a variety of age-related diseases hold great promise as critical indicators for early diagnosis and treatment of age-related diseases.

## Senescence-associated secretory phenotype (SASP) in the skeletal system

SnCs secrete dynamic bioactive components, including soluble signalling factors, chemokines, inflammatory cytokines, oxidative stress-related proteins, proteases, and an insoluble protein/extracellular matrix (46). SASP is one of the most prominant features of SnCs and displays multiple functions. The pro-apoptotic SASP causes a series of detrimental effects in organisms through autocrine and paracrine signalling (46).

SASP factors are crucial regulators of senescence, thereby driving systemic chronic inflammation characterised by the maintenance of low-grade sustained inflammatory responses even in the absence of acute infection or overt clinical disease (47). A sustained inflammatory environment can impair the function of neighbouring and distant non-SnCs, contributing to cell aging and initiation of age-related chronic diseases (48).

SASP has been well involved in bone remodelling and degenerative diseases of the skeleton. The SASP in aging bone tissues is mainly produced by senescent osteocytes and myeloid progenitors in humans and mice. SASP-related cytokines, including interleukin-6 (IL-6), IL-8, monocyte chemotactic protein 1 (MCP-1), and tumour necrosis factor α (TNF-α), are substantially upregulated in the osteocytes of aged mice (49). Senescent osteocytes inhibit osteogenesis and promote osteoclastogenesis via SASP factors (50, 51). In OA, senescent chondrocytes secrete SASP factors that impair cartilage function (52). In addition to skeletal cells, immune cells also undergo senescence to produce SASP factors in the bone microenvironment. The crosstalk between inflammatory immune cells and senescent MSCs deteriorates SnCs (53).

Because the SASP includes a series of inflammatory factors, it is difficult to identify its specific role in the bone microenvironment. Among the SASP components, IL-6 is a possible candidate for a relevant inflammatory factor in age-related bone loss (54). In an obesity model, IL-6 accelerated BMSCs senescence via the IL-6/STAT3 signalling pathway. IL-6 knockout prevents BMSCs senescence and alleviates obesity-induced bone loss (55). Additionally, IL-6 may suppress Setd7 expression via the Akt pathway and impair MSC osteogenesis (56). In contrast, senescent BMSCs robustly produced IL-6. IL-8, another important member of the SASP family, exhibits an expression pattern similar to that of IL-6. Chondrocytes from patients with OA highly express both IL-6 and IL-8, whereas forkhead box O (FoxO) 3a knockdown reduces IL-6 and IL-8 production (57). Notably, IL-6 levels are correlated with the senescence index (58). Although the relationship between SASP and aging-associated bone disorders has received extensive attention, more research is needed to identify the molecular mechanisms of SASP in bone homeostasis regulation, which will provide potential targets for therapeutic intervention in skeletal diseases.

## Senescence-associated signalling in the skeletal system

Bone development and regeneration rely on the coordination between multiple signalling pathways that mediate bone remodelling. Aging leads to the dysfunction of its own signalling, further affecting the stability of the skeletal system. Although our understanding of the regulation of senescence in aged skeletal system remains incomplete, several factors have been identified. Additionally, several signalling pathways participate in the regulation of senescence in age-related bone loss.

### Hedgehog (HH) signalling

HH is a protein secreted via paracrine signallying that functions as a morphogen. The HH pathway is evolutionarily conserved and includes three main HH members in mammals: sonic hedgehog (Shh), Indian hedgehog (Ihh), and desert hedgehog (Dhh) (59). The HH pathway is primarily activated by two multispan transmembrane proteins: smooth (Smo) and patch 1 (Ptch1). When bound to its receptor, Ptch1, HH represses SMO, thus activating downstream gene transcription (60). Many studies have demonstrated the critical role of HH signalling in bone formation. Shh signalling promotes osteoblastic differentiation of MSCs by upregulating alkaline phosphatase, osteocalcin, and runt-related transcription factor 2 (*Runx2*) gene expression. Ihh induces chondrogenesis in human MSCs and is involved in cartilage and bone formation. Interestingly, the downregulation of Ihh in chondrocytes efficiently reduces OA progression (61).

In recent years, HH signalling has been shown to be closely associated with cellular senescence and age-related diseases, including Alzheimer's disease, skin aging, and cardiovascular diseases (62). Shh directly stimulates osteoclastic formation and delays fracture healing in aged mice (59, 63). Senescent MSCs show increased expression of GATA-binding protein 6 (GATA6), which inhibits downstream Shh signalling to induce cellular senescence and senescence-related activities (64). Hydrogen peroxide-induced oxidative stress inhibits HH signalling in M2-10B4 MSCs and C3H10T1/2 cells, leading to suppressed osteogenic differentiation (65, 66). Exogenous Ihh treatment rescues BMSCs from senescence by suppressing the ROS/mTOR/4EBP1 and p70S6K1/2 pathways (67). This evidence highlights that HH signalling can regulate bone formation by regulating MSCs senescence in age-related diseases.

### Notch signalling

The Notch signalling pathway also determines cell fate and function in the skeletal system. Four Notch receptors (Notch 1–4), Jagged-1 and -2, and Delta-like-1/3/4, have been identified at the cell membrane. Upon ligation, the Notch intracellular structural domain (Nicd) is activated and induces associated gene transcription to regulate bone formation. Similar to HH signalling, Notch signalling is crucial for the maintenance of bone homeostasis and regulation of aging. Deficient Notch signalling is accompanied by progressive bone loss in old mice (68, 69). Activation of Notch signalling by Jag1 and Hes1 alleviates BMSCs senescence, thereby enhancing the regenerative capacity of MSCs (70). Nicd overexpression can protect bones from loss and enhance bone healing in aging and ovariectomized mice (71). Since strong blood flow can activate Notch activity in aged mice, Notch activity is inhibited in the endothelium due to reduced blood flow, leading to decreased osteogenesis (69).

### Wnt/β- catenin signalling

The Wnt/β-catenin pathway is involved in tissue development and various other physiological progress (72). Wnt signalling can be classified into the classical Wnt/β-catenin pathway and the nonclassical Wnt pathway. Although Wnt signalling-related proteins are substantially downregulated in the myeloid nuclei of postmenopausal women, aged mice, and ovariectomized rats (73–77), the mechanism underlying this process remains unclear. Wnt/β-catenin signalling maintains bone homeostasis by antagonising MSCs senescence and inhibiting SASP (78). Secreted Frizzled-Related Protein 4 (sFRP4) is an extracellular Wnt antagonist, and sFRP4 gene interference can activate the Wnt/β-catenin signalling pathway and relieve age-related bone loss (79). Systemic inhibition of RhoA/Rock signalling results in the activation of Wnt/β-catenin signalling, leading to reduced bone loss during aging. Elevated levels of miR-1292 can inhibit the Wnt/β-catenin signalling pathway, leading to enhanced senescence of MSCs, thereby hindering their differentiation into osteoblasts (80). In addition, age-related mechanical loading inhibits bone formation, which may be attributed to impaired Wnt signalling activation (81–83). In summary, Wnt/β -catenin signalling regulates bone homeostasis. Further studies are needed to clarify the mechanism by which Wnt/β-catenin participates in bone cell senescence and age-related diseases.

### Transforming growth factors (TGF)/bone morphogenetic protein (BMP) pathway

Notably, it is well-accepted that highly conserved BMP signalling can regulate bone formation through engagement with TGF-β/BMP superfamily ligands, BMP receptors, and signalling sensors (84). BMP2, currently the only osteogenic factor approved by the FDA for clinical use, promotes osteoblast differentiation by targeting *Runx2* (85). We found that silencing miR-106b promotes the osteogenesis of BMSCs and alleviates OP induced by glucocorticoids via targeting BMP2. Moreover, the myokine irisin regulates osteogenesis by activating BMP/SMAD signalling via αV integrin (86). Our work further confirmed TGF/ BMP signalling is revelent to osteogenesis and bone formation from new points (87).

Recently, osteocyte transcriptomes showed that BMP was an age-related signalling pathway, as evidenced by decreased BMP signalling in BMSCs from aged or osteoporotic subjects (88). BMP 2/4 is essential for bone homeostasis, as it regulates Smad6 expression. With aging, Smad6 expression gradually decreases, indicating the impaired function of BMP 2/4 signalling and its involvement in bone loss (89). In contrast, there is an increased level of pleckstrin homology domain-containing family O member 1, a molecule related to ubiquitination that inhibits BMP signalling, leading to decreased bone formation during aging (90).

### Fibroblast growth factor (FGF)

The FGF family is comprised of 22 members in humans and mice. When FGFs interact with the specific membrane tyrosine kinase FGF receptor (FGFR), they phosphorylate tyrosine residues in the intracellular structural domain of FGFR, activating downstream signalling pathways such as MAPK, PI3K-Akt, and PKC (91, 92). FGF signalling and its receptors are essential for all steps of skeletal development and osteoblastic differentiation (93, 94). The FGF family has been shown to stimulate self-renewing proliferation and prevent cellular senescence in certain tissues, and three of these factors (FGF2, FGF18, and FGF23) have received increasing attention. Thus, FGF2 may stimulate the proliferation of aging osteoblast progenitor cells. Additionally, FGF2 substantially increases osteoblast mineralisation in aged rats, indicating that FGF2 may have a therapeutic effect on age-related diseases (95). FGF18 is currently being tested for the treatment of OA (96). FGF23 is mainly produced by the skeletal system, which is different from FGF2 and FGF18 production. FGF23 and its receptor Klotho protein, participate in the aging process by regulating phosphate homeostasis, vitamin D metabolism, Wnt, and the p53/p21 signalling pathway, which are involved in the pathophysiology of osteoporosis and vascular calcification (97).

## Age-related skeletal diseases

### Osteoporosis (OP) and fracture healing

Decreased bone mass and bone tissue microarchitecture deterioration are characteristic features of OP, whcih is the most common metabolic bone disease that leads to an increased risk of fragility fractures (98). Although OP can occur in all age groups, primary OP is considered an age-related disease. Postmenopausal women and older men are more likely to develop OP due to sex steroid deficiency and aging. In addition, medications and other diseases can induce OP (secondary OP). OP is mainly caused by an imbalance in bone remodelling, which involves bone formation by osteoblasts and resorption by osteoclasts (99). BMSCs, osteocytes, osteoblasts, and osteoclasts are the major cells involved in bone remodelling. Senescent BMSCs differentiate into adipocytes more than osteoblasts. With aging or other stimuli, senescent osteocytes accumulate in the bone and disrupt the osteogenic differentiation of BMSCs through the SASP (100). Impressively, there was no substantial difference in the levels of aging markers between BMSCs from osteoporotic (OP-BMSCs) and healthy rats (H-BMSCs); however, the anti-aging ability of OP-BMSCs was substantially decreased by intervention with H_2_O_2_ (101).

**Fig. 1:**
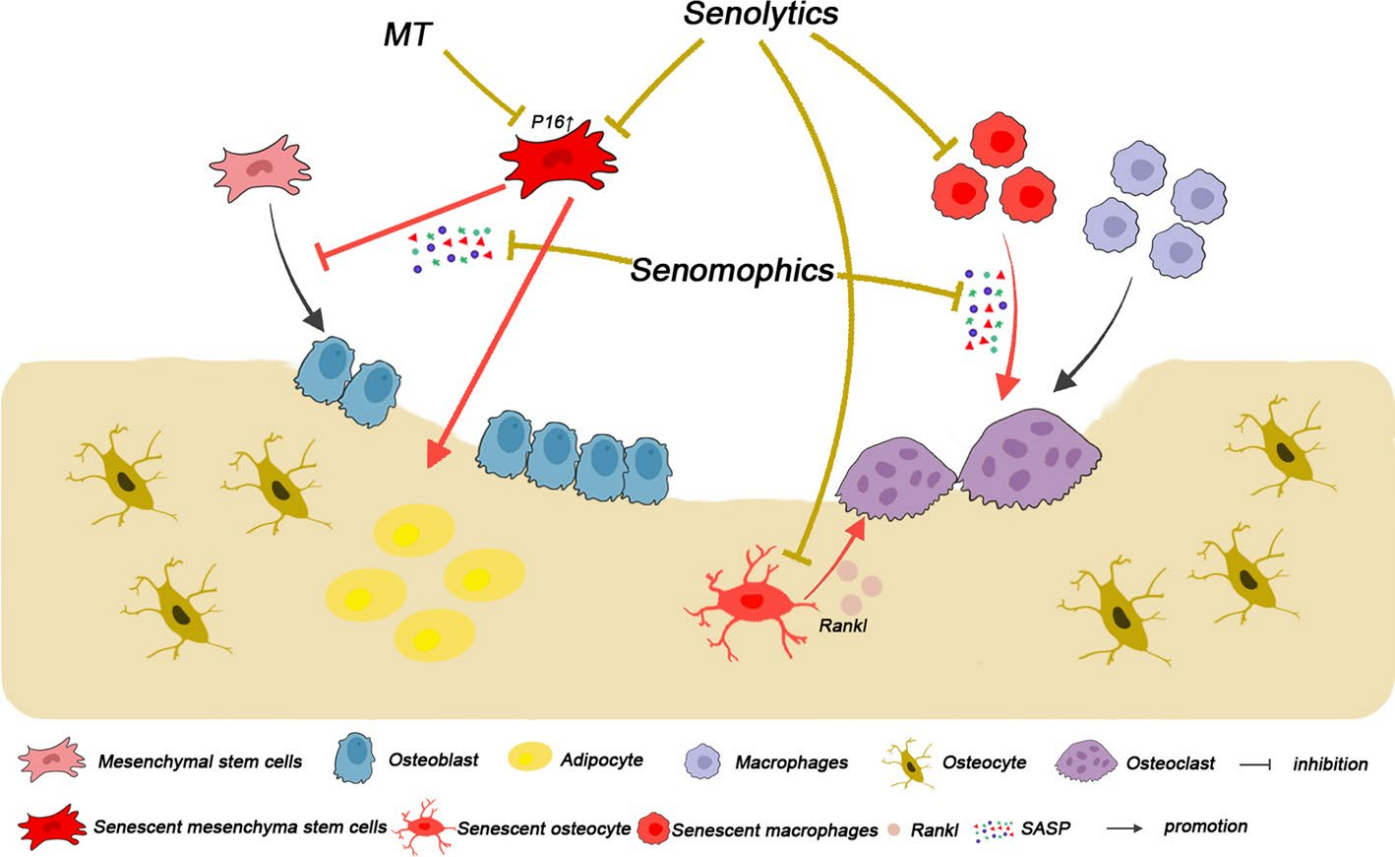
Strategies targeting cellular senescence in osteoporosis. Senescent MSCs (red arrows) can inhibit the osteogenic differentiation and promote adipogenic differentiation of physiologic MSCs. Senescent macrophages (red arrows) can enhance osteoclast activity. Senescent osteocytes secrete high levels of RANKL to promote osteoclast activity leading to increased bone loss. A common feature of SnCs is the development of SASP. "Senolytics" and "senomophics" (yellow arrows) are necessary to counteract the adverse effects of SnCs by killing SnCs and inhibiting SASP secreted by senescent cells, respectively

Glucocorticoids (GC) and chemotherapy (doxorubicin) are common causes of secondary OP and can induce senescence in several cell types, including blood vessel cells, endothelial cells, MSCs, and osteoblasts, contributing to bone loss. Although chemotherapy is an efficient cancer treatment, it has direct side effects on bone remodelling. Chemotherapy increases p16 and p21 expression in CD45^−^CD31^−^ bone-resident cells. Senescent immune cells, such as macrophages and neutrophils, secrete granulocalcin, which inhibits osteogenic differentiation of BMSCs (102). SnCs and SASP eventually lead to rapid bone loss and ultimately, osteoporosis (103) (Fig. 1).

MiRNAs are key regulators of bone metabolism during aging (104). Moreover, MiR-146a expression is positively associated with aging. MiR-146a deficiency protects mice from ovariectomy-induced bone loss, suggesting that downregulation of miR-146a may be a novel target to counteract age-dependent bone loss (105). Additionally, miRNAs can transmit senescence signals through intercellular communication (106). MiR-183-5p levels in bone-derived Extracellular Vesicles (EVs) in the bone marrow increase with age and miR-183-5p inhibits BMSC proliferation and promotes cellular senescence in stem cells (107). Substantially increased miR-34a levels in the EVs of skeletal muscle and serum from aged mice reduced BMSCs viability and promoted cellular senescence within the bone marrow microenvironment (108). MiR-20a is down-regulated in the elderly individuals (109); however, miR-20a delivery directly promotes the osteogenic differentiation of BMSCs (110). MiR-19a-3p and miR-21-5p are senescence-associated miRNAs that promote the restoration of BMSC activity (111, 112).

Increased bone fragility due to senile OP increases the risk of fractures (113) and aging delays fracture healing (114). Stem cells self-renew, persist throughout life, and are responsible for tissue homeostasis. They replace damaged cells in response to injury, disease and aging (115). Aging leads to the loss of stem cell activity in the bone and deterioration of the bone marrow microenvironment, thereby prolonging the healing time of osteoporotic fractures (116). Increased SASP in the somatic circulation of aged mice reaches the bone marrow microenvironment and delays bone formation and fracture repair by impairing skeletal stem cell function (117). Increasing age in mice leads to reduced blood vessel formation and bone regeneration in fracture-healing tissue (118). Transplantation of BMSCs from aged mice reduced bone formation at the site of cranial defects in young mice compared to transplantation of BMSCs from young mice, and the reduced bone-formation capacity correlated with reduced Wnt signalling in aged BMSCs (119). Our previous study confirms these findings (75). In addition, fractures in aged mice lead to the accumulation of SnCs at the fracture site, which inhibits the viability of MSCs through the expression of TGF-β1, thereby delaying fracture healing (120). Stem cell dysfunction and age-related deterioration of the bone marrow microenvironment at the fracture site affect fracture healing (121). Therefore, understanding the relationship between aging and bones is critical for improving osteoporosis and fracture healing.

## Osteoarthritis (OA)

OA is a prevalent degenerative joint disease characterised by joint inflammation, cartilage damage, and joint pain (122). In OA, SnCs can be detected in joint structures, such as articular cartilage, synovium, and subchondral bone, suggesting that SnCs have a detrimental effect on the occurrence and development of OA (123). SASP factors including IL-1β, IL-6, TGF-β, TNF-β, and matrix metalloproteinases 3/13 (MMP3/13), can also observed in these joint structures (12, 124). The persistent presence of a SASP aggravates chronic inflammation and eventually aggravates OA. IL-6 promotes the secretion of SASP factors, which, in turn, reinforces senescence. Male IL-6^−/−^ mice exhibited severe joint injuries in OA (125).

**Fig. 2:**
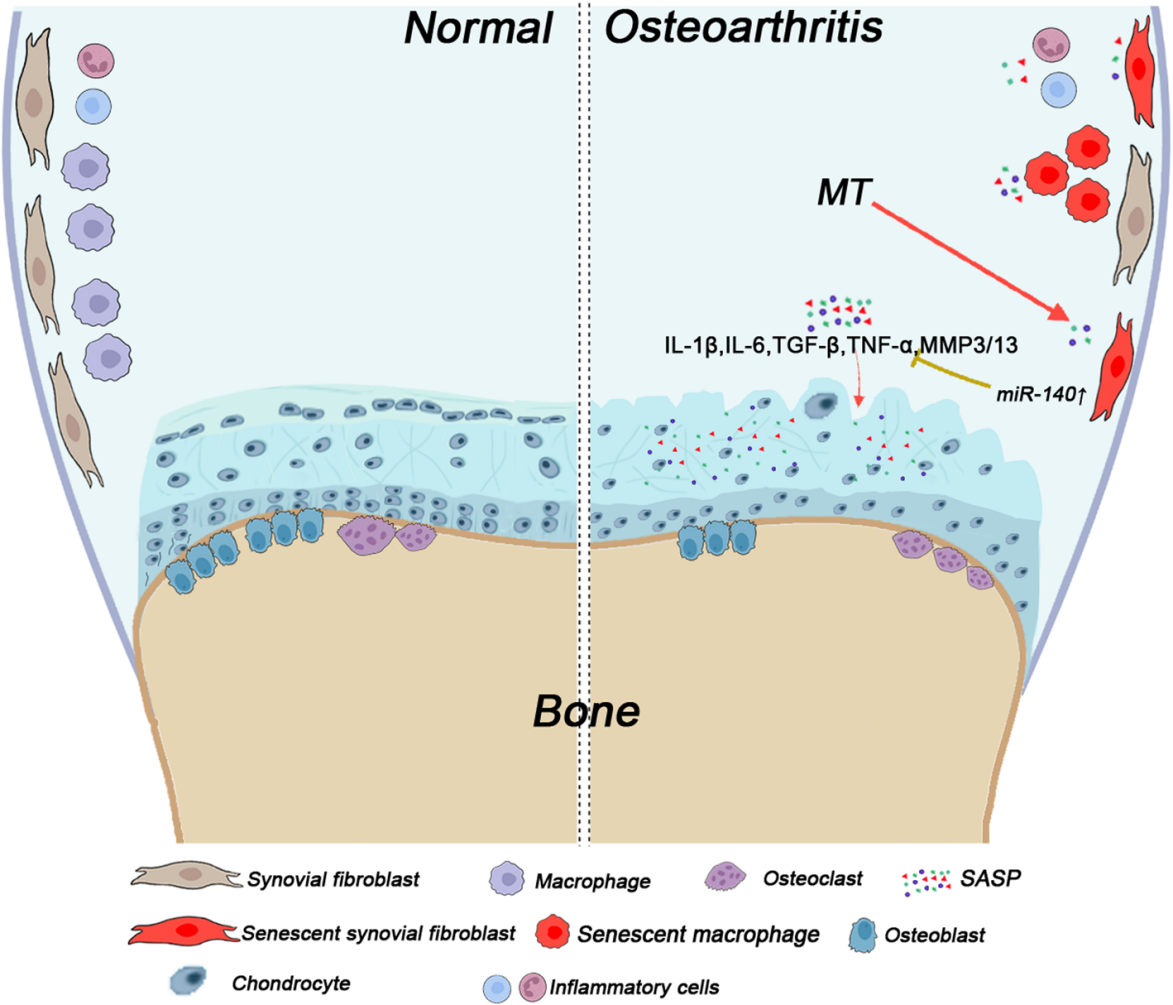
Strategies targeting cellular senescence in osteoarthritis. In OA, various cells can secrete SASP components. When SnCs persist, molecules secreted by SnCs and immune cells can cause chronic inflammation, eventually leading to OA

Autophagy is critical for preserving the function of articular cartilage; however, it decreases with age (126). METTL3-mediated m6ATG7 modification promotes fibroblast-like synoviocyte senescence and OA development by regulating autophagy (127). Activation of PI3K/Akt/mTOR signalling suppresses autophagy and mediates chondrocyte cellular senescence, which is responsible for disorders of cartilage homeostasis and integrity in OA (128, 129). The sirtuin family plays an important role in preventing OA progression. Our group found that Sirt3 in the mitochondria alleviates mitochondrial dysfunction and chondrocyte senescence, mainly by regulating the deacetylation of the cytochrome c oxidase subunit 4 isoform 2 (130). Overexpression of Sirt6 dramatically rescues chondrocytes from senescence by suppressing IL-15/JAK3/STAT5 signalling (131). Although the mechanisms by which SnCs and SASP factors contribute to OA are still being investigated, targeting senescence has proven to be a promising strategy for OA treatment (Fig. 2).

## Intervertebral disc degeneration (IDD)

The intervertebral disc (IVD) is a fundamental component of the spine that contributes space and connects adjacent vertebrae. The IVD consists of three distinct components: the outer annulus fibrosus (AF), central gelatinous nucleus pulposus (NP), and the cartilage endplate (CEP), which is anchored to the vertebral body. IDD is a common degenerative disease of the skeletal system and is a leading cause of chronic low back pain in the elderly. Unlike other soft tissues, intervertebral disc cells are chronically exposed to a unique microenvironment characterised by limited nutrient supply, hypoxia, hypertonicity, low potential of hydrogen, and various mechanical loads (132). During aging, IDD undergoes pathological changes, including fibrosis of gelatinous NP tissue, transformation of vacuolar notochord cells into chondrocyte-like NP cells, thinning of the endplate cartilage layer, disintegration of the AF, and fibrocartilage metaplasia (132). Moreover, many senescent disc cells accumulate in the IVD (133). SASP factors secreted by senescent disc cells can alter gene expression from anabolic to catabolic pathways, leading to an increase in the secretion of matrix-degrading enzymes. SASP also promotes senescence in IVD cells through paracrine signalling, leading to IDD and intervertebral disc-derived low back pain (134). In addition, immune cells in IVD secrete a series of inflammatory mediators (TNF-α, IL-6, and Prostaglandin E2), which reduce the number of NP cells and deteriorate the local microenvironment (135). IL-1 promotes senescence of NP cells and plays a crucial role in the pathogenesis of disc degeneration. IL-1^−/−^ knockout mice develop similar characteristics of human disc degeneration. Several cell signalling pathways have been identified in disc cell senescence (136). The activation of Wnt/β-catenin signalling can increase MMP and TGF expression in NP cells and promote cellular senescence (137). Sirtuin 1 (SIRT1) regulates p53 deacetylation in human NP cells (138) and reduces p16 expression in mouse disc cells (139), thus reducing cellular senescence and alleviating IDD. In human NP cells, mechano-stress can trigger nuclear factor-kappa B activation, resulting in senescence and periostin secretion to induce IDD (140).

## Strategies targeting cellular senescence

As previously mentioned, many SnCs are involved in age-related skeletal diseases. Removing SnCs from the skeletal system is critical for slowing skeletal aging and alleviating disease. In INK-ATTAC or p16-3MR transgenic mice, drugs targeting p16^Ink4a^ successfully remove p16^Ink4a^-positive SnCs, alleviating age-related bone loss and extending the lifespan of mice (141, 142). Targeting cellular senescence has led to the emergence of "senolytics" (selectively eliminating SnCs) and "senomophics" (inhibition of SASP secretion) to neutralize and eliminate the adverse effects of SnCs, which are collectively referred to "senotherapeutic" strategies (143).

### SnCs clearance

Several "senolytic" compounds are identified to possessthe potential to kill SnCs (144). Senolytic targets include both anti-apoptotic and pro-survival pathways, such as the B-cell lymphoma-2 protein family, PI3K, Akt, p53, p21, serpins, epinephrine, tyrosine kinase, hypoxia-inducible factor-1α, heat shock protein 90, FoxO4, and histone deacetylases (145). Melatonin (MT) reverses H_2_O_2_-induced aging of BMSCs by decreasing the expression of the senescence-associated protein p16^INK4α^ and increasing the expression of *Sirt1* (146, 147) (Fig. 1). Targeted p21 depletion prevents DNA damage-induced osteoporosis and increases bone marrow adiposity in mice (148). Dasatinib combined with quercetin and quercetin alone is known to eliminate SnCs from the skeletal system, thereby reducing bone loss (149). Drugs with senolytic activity are widely used. Fisetin is a natural flavonoid polyphenol with a senolytic activity. Fisetin administration reduces the levels of senescence markers (150). In OA treatment, MT substantially upregulated the expression of miR-140 (151), which is considered a key participant in the pathophysiology of age-related diseases (152) (Fig. 2). To date, an increasing number of senolytics are in various stages of development for clinical use. Targeting SnCs with senolytics has the potential to treat age-related skeletal disorders. In practice, enhancing microvessel density in skeletal muscles by restoring vascular endothelial growth factor levels is beneficial for alleviating the development of sarcopenia and osteoporosis in aging individuals (153). Studies have demonstrated that rejuvenating aging individuals by locally targeting SnCs in the skeletal system can reverse aging of the whole body, thereby representing a new promising therapeutic strategy.

### SASP reduction

The SASP facilitates the progression of inflammation and accelerates aging. Neutralizing/inhibiting the SASP is an alternative strategy to delay aging. Lamivudine, a nucleoside reverse transcriptase inhibitor, inhibits type-I interferon (IFN-I) activation and reduces inflammation by inhibiting SASP in aged mice (154). Inhibitors of IL-1β, TNF-α, and IL-6 have been demonstrated to be efficacious in treating inflammatory arthritis. Neutralising or inhibiting the SASP reduces the clinical symptoms of inflammation and pain in OA (Fig. 2). Thus, the JAK/STAT pathway is involved in regulating cytokine production, and the JAK inhibitor ruxolitinib inhibits secretion of SASP factors (IL-6, IL-8, and PAI-1) and alleviates age-related bone loss (52). Although the reduction in SASP does not directly address the underlying causes of inflammation and tissue degeneration, it presents a promising approach for boosting bone density in diseases related to aging.

### Stem cell therapy

Stem cells possess the unique ability to self-renew and differentiate into multiple lineages, making them therapeutically attractive for regeneration. Stem cells, particularly MSCs, have been widely used to treat skeletal diseases, including osteoporosis and OA. Intra-bone marrow injection of MSCs efficiently alleviates bone loss in osteoporotic rats (155). We administrate BMSCs to delay matrix degeneration in NP cells by downregulation of SASP-associated NF-κB signalling in IDD mice (156). Recently, stem cell-derived EVs were shown to exert significant effects on tissue repair and regeneration. EVs are small vesicles released by almost all cell types and mediate signal transmission between cells. EVs derived from umbilical cord MSCs (UC-EVs) effectively rejuvenated senescent BMSCs by enhancing telomere length and viability. Moreover, UC-EVs improve bone formation in aging mice by transferring proliferating nuclear antigens to receptor cells (157). EVs produced by hypothalamic stem cells regulate aging via miRNAs (158). Adipose MSC-derived EVs decrease senescence markers and promote muscle and kidney tissue repair (159). In summary, stem cells and EVs can reduce cell senescence, combat skeletal aging, and reduce age-related diseases.

### Specific clearance of SnCs

Chimeric antigen receptor (CAR)-T cell therapy has been successful in tumour therapy. In 2017, the FDA approved the use of anti-CD19 CAR T-cell therapy for refractory aggressive non-Hodgkin's in humans (160). Therapeutic CAR-T cells targeting SnCs have been proposed as "senolytics." Studies have shown that urokinase fibrinogen-activated receptor (uPAR) -specific CAR-T cells can effectively eliminate SnCs, both in vitro and in vivo (161). uPAR is a transmembrane protein that is widely expressed during senescence. Therefore, identifying a cell surface protein that is widely upregulated in SnCs for designing CAR-T cells may be of great importance in the treatment of age-related diseases.

Proteolysis-targeting chimeric (PROTAC), a bivalent small molecule composed of ligands, is an emerging drug discovery platform with the potential to reduce the need for drug exposure (162). PROTAC recruits E3 ligases and ubiquitinates target proteins (163). PZ15227 transformed by ABT263 was found to be more effective than ABT263 in clearing SnCs from aged mice (162). Another study showed that PROTAC, another ARV825 molecule consisting of PROTAC has significant senolytic activity (164). In a mouse model of obesity-induced hepatocellular carcinoma, ARV825 treatment eliminated SnCs from the liver and delayed the development of hepatocellular carcinoma (164).

As mentioned above, an abnormal increase in lysosomal SA-β-gal activity is a characteristic of SnCs. Although SA-β-gal is not a specific marker of senescence, senescent tissues are typically positive for it (165). Muñoz-Espín et al. designed a targeted delivery system for galactose oligosaccharide-coated porous silica particles. This delivery system eliminates cells with high SA-β-gal activity (166). Xiong et al. synthesised an β-galactosidase-responsive photosensitiser that was selectively activated by SA-β-gal in SnCs, thus effectively killing cells by photodynamic action (167). β-Gal-modified prodrugs are another novel anti-aging strategy that can be specifically cleaved by lysosomal SA-β-gal into fragments with anti-aging effects to ameliorate the aging conditions of the organism (30). The synthesis of senescence-specific killing compounds by gemcitabine and β-gal releases gemcitabine in the presence of high SA-β-gal activity, thereby eliminating SnCs (168). However, in addition to SnCs, a significant proportion of normal cells also express β-gal, and these cells can be eliminated by β-gal-targeted delivery systems and β-gal prodrug systems (168).

## Conclusion and future outlook

Aging is associated with the skeletal system. SnCs and the SASP have critical effects in a variety of clinical diseases. Although the exact role of SnCs in skeletal diseases during aging needs to be further investigated, the "senotherapeutic" strategy has given us hope to slow or reverse aging. This strategy effectively removes SnCs and eases the aging symptoms of related diseases; however, it brings about new problems such as the requirement for long-term use and the side effects of long-term use. Therefore, a proper understanding of the mechanism of action of SnCs in skeletal diseases and the development of novel anti-aging strategies or the reduction of the side effects of existing anti-aging drugs are of great significance for the future treatment of age-related diseases other than orthopaedic diseases.

## Data Availability

Not applicable.

## Abbreviations

OA: Osteoarthritis
SASP: Senescence-associated secretory phenotype
DDR: DNA damage response
HGPS: Hutchinson-Gilford progeria syndrome
WS: Werner syndrome
ROS: Reactive oxygen species
SA-β-gal: Senescence-associated β-galactosidase
MiRNAs: MicroRNAs
IL-6: Interleukin-6
MCP-1: Monocyte chemotactic protein 1
TNF-α: Tumor necrosis factor α
BMSCs: Bone marrow mesenchymal stem cells
MSCs: Mesenchymal stem cells
FoxO: Forkhead Box O
Shh: Sonic hedgehog
Ihh: Indian hedgehog
Dhh: Desert hedgehog
Smo: Smooth
Ptch1: Patch 1
Runx2: Runt related transcription factor 2
GATA6: GATA-binding protein 6
mTOR: Mammalian target of rapamycin
MiDAS: Mitochondrial dysfunction-associated senescence
Nicd: Notch intracellular structural domain
sFRP4: Secreted Frizzled Related Protein 4
OP-BMSCs: BMSCs from ovariectomized-osteoporotic rats
H-BMSCs: BMSCs from health rats
miR: MicroRNA
TGF: Transforming growth factors
BMP: Bone morphogenetic protein
FGF: Fibroblast growth factor
FGFR: FGF receptor
GC: Glucocorticoid
EVs: Extracellular vesicles
MMP3/13: Matrix metalloproteinases 3/13
IDD: Intervertebral disc degeneration
IVD: Intervertebral disc
AF: Annulus fibrosus
NP: Nucleus pulposus
CEP: Cartilage endplate
SIRT1: Sirtuin 1
MT: Melatonin
IFN-I: Type-I interferon
UC-EVs: EVs derived from umbilical cord MSCs
CAR: Chimeric antigen receptor
PROTAC: Proteolysis-targeting chimeric
uPAR: Urokinase fibrinogen-activated receptor
